# Impact of a Maternal Early Warning System and Severe Hypertension Safety Bundle on Timely Treatment of Hypertensive Emergencies: A Quality Improvement and Health Equity Initiative

**DOI:** 10.1111/1471-0528.18273

**Published:** 2025-07-17

**Authors:** Towana D. Sims, Lauren Shubert, Stacie Denning, Lena Shay, Celeste Green, Christina Davidson

**Affiliations:** ^1^ Department of Obstetrics and Gynecology Texas Children's Hospital Houston Texas USA; ^2^ Department of Obstetrics and Gynecology, Division of Maternal‐Fetal Medicine Baylor College of Medicine Houston Texas USA

**Keywords:** disparity, hypertensive emergency, maternal early warning system (MEWS), patient safety bundle, severe hypertension

## Abstract

**Background:**

Hypertensive disorders of pregnancy are a leading cause of pregnancy‐related morbidity and mortality. Timely treatment of a hypertensive emergency has the potential to reduce preeclampsia‐related morbidity and mortality.

**Objective:**

To evaluate the impact of quality improvement and patient safety initiatives on timely treatment of hypertensive emergencies in pregnancy and racial and ethnic disparities.

**Setting:**

Maternity teaching hospital in Texas Medical Center.

**Population:**

Pregnant and postpartum women with hypertensive emergencies.

**Methods:**

A retrospective chart review of all hospital deliveries with a hypertensive emergency was conducted from 1 January 2017 through 31 December 2024. During the study period, our hospital implemented a maternal early warning system (MEWS) and a patient safety bundle for severe hypertension (SHTN) in pregnancy. All deliveries ≥ 20 0/7 weeks were included.

**Main Outcomes Measures:**

Rate of timely treatment of SHTN, overall and by race and ethnicity.

**Results:**

There was a statistically significant improvement in the rate of timely treatment of SHTN with implementation of MEWS (69.5% at baseline/phase 1 vs. 79.9% with MEWS/phase 2, *p* < 0.001, 15% improvement) and then again with implementation of the SHTN bundle/phase 3 (88.8% in phase 3, 11% improvement compared to phase 2 and 27.8% improvement compared to phase 1, *p* < 0.001). Changes over time within each racial and ethnic group indicated that the rate of timely treatment of a hypertensive emergency increased significantly for NH‐Black, NH‐White and Hispanic patients from phase 1 to phase 2 and for all groups from phase 1 to phase 3. The disparity in treatment fallouts and timely treatment was eliminated with MEWS modifications and the patient safety bundle in the final phase.

**Conclusion:**

Quality improvement and patient safety efforts that standardise response to and treatment of hypertensive emergencies can lead to improvement in timely treatment and elimination of racial and ethnic disparities.

## 1 Introduction

Hypertensive disorders of pregnancy represent 6.8% of all pregnancy‐related deaths in the United States [1]. The prevalence of hypertensive disorders of pregnancy among delivery hospitalisations increased from 13.3% to 15.9% during 2017–2019, and 31.6% of maternal deaths that occurred during a delivery hospitalisation had a hypertensive disorder documented [2]. The Texas Maternal Mortality and Morbidity Review Committee (MMMRC) and Department of State Health Services (DSHSs) Joint Biennial Report found that preeclampsia‐associated severe maternal morbidity (SMM) rates increased 37% between 2017 and 2020 in Texas [3]. From 2019 to 2020, rates remained stable among non‐Hispanic (NH) White populations, declined in NH‐Other populations, and increased among NH‐Black and Hispanic populations. The report also found that 90% of pregnancy‐related deaths were noted to have some chance of preventability [3]. Among 54 preeclampsia pregnancy‐related deaths that occurred in California from 2002 to 2007, 33 were attributed to stroke [4]. Systolic blood pressure exceeded 160 mmHg in 96% of cases, and diastolic blood pressure was 110 or higher in 65% of cases, with only 48% of those patients receiving antihypertensive treatment [4]. A good‐to‐strong chance to alter outcome was identified in 66% of stroke cases, with delayed response to clinical warning signs in 91% of cases and ineffective treatment in 76% of cases being the most common areas for improvement [4].

The American College of Obstetricians and Gynecologists (ACOG) defines acute‐onset severe hypertension (SHTN) (i.e., hypertensive emergency) as systolic blood pressure of 160 mmHg or more, and/or diastolic blood pressure of 110 mmHg or more, that is confirmed as persistent (15 min or more), and recommends that antihypertensive agents be administered within 30–60 min of persistent SHTN [5]. Recent reports, however, demonstrate a quality gap between the recommended expeditious treatment of SHTN and the actual performance. In several studies, the treatment of SHTN within 60 min was initiated in less than one‐half of cases [6]. Maternal early warning systems (MEWS) have been proposed to facilitate timely recognition, diagnosis and treatment for women developing critical illness and may be a pathway to ensuring timely treatment of a hypertensive emergency [7].

The Alliance for Innovation on Maternal Health (AIM) has a patient safety bundle for SHTN in pregnancy in an effort to reduce its associated morbidity and mortality [8]. The readiness and recognition domains include having standards for early warning signs as well as a standard response to them [8]. Our hospital implemented a MEWS in June 2018. In 2021, Texas DSHS launched a statewide initiative to implement an adapted version of the AIM SHTN patient safety bundle (TexasAIM) and invited all Texas birthing hospitals to participate. TexasAIM's adaptation incorporated disparity‐focused structure, process and outcome measures, given the known disparities in hypertension‐associated maternal mortality and morbidity in Texas, with NH Black women having the highest rates [3]. The primary process measure for bundle implementation was the rate of timely treatment of a hypertensive emergency. Since much of the morbidity and mortality associated with hypertensive disorders of pregnancies is related to SHTN, our project sought to evaluate the impact of our MEWS combined with the implementation of the SHTN patient safety bundle on the TexasAIM process metric of timely treatment of a hypertensive emergency in pregnancy and any associated racial and ethnic disparities. Our focus on racial and ethnic disparities aligned with the 2022 Texas MMMRC's recommendation for implementation of statewide maternal health and safety initiatives with incorporation of health equity principles to reduce maternal mortality, morbidity and health disparities [3].

## 2 Methods

We report on our study design, data analysis and outcomes following the Standards for Quality Improvement Reporting Excellence (SQUIRE) 2.0 [9]. Our hospital is a level IV maternal and neonatal hospital located in an urban medical centre. This designation is assigned by the Texas DSHS and indicates that our hospital provides the highest level of comprehensive care for pregnant and postpartum patients [10]. With an annual delivery volume of over 6500 births, our hospital is staffed by both academic and private practise obstetric physicians and certified nurse midwives (CNMs) and supports the training of one of the largest obstetrics and gynaecology residency programmes in the United States.

**FIGURE 1:**
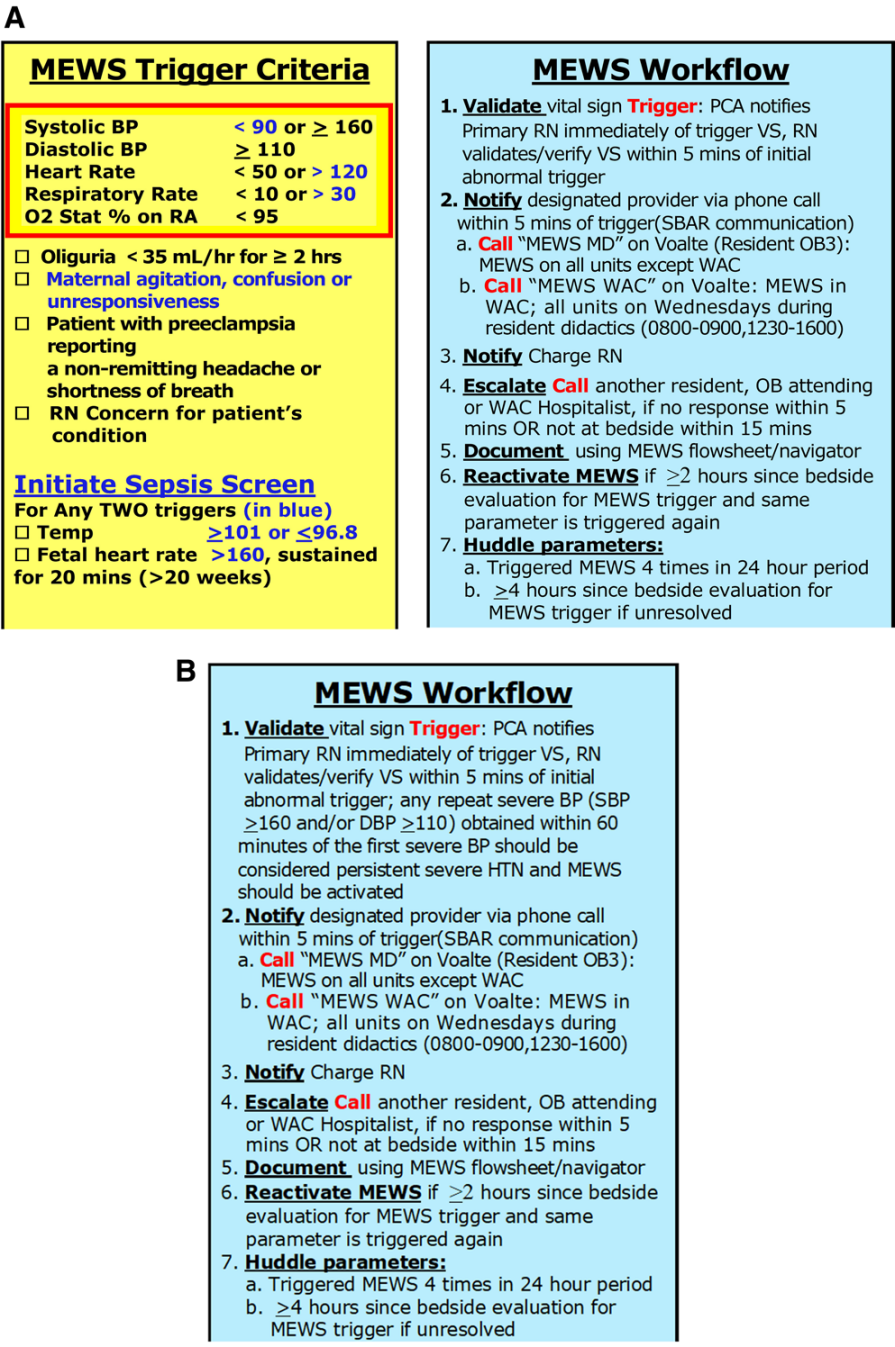
Maternal early warning system (MEWS) badge with MEWS trigger criteria and MEWS workflow. (A) MEWS workflow through 2023. (B) MEWS workflow beginning in 2024. Beginning in January 2024, the process was updated to activate a MEWS response for any severe blood pressure obtained within 60 min of the first severe blood pressure, even if interspersed with non‐severe blood pressures.

Our hospital has previously reported on our experience and outcomes with the implementation of the obstetric haemorrhage bundle with TexasAIM, and we adopted the same model for the implementation of the SHTN bundle [11]. In addition to forming hypertension workgroups, we engaged our pre‐existing hospital MEWS workgroup, which continued to audit our MEWS process as part of our sustainability efforts from that patient safety initiative. We adapted our MEWS process from the maternal early warning criteria set forth by the National Partnership for Maternal Safety, employing a single‐parameter system [7]. Any vital sign that meets MEWS criteria should be verified within 5 min by a registered nurse to exclude erroneous measurements. If the vital sign aberration persists, a designated MEWS provider (senior level resident, faculty, or CNM) is notified via phone call within 5 min of the trigger. The provider is expected to present to the bedside within 15 min for in‐person evaluation. There is an established escalation system if the assigned MEWS provider cannot be reached or is unable to evaluate the patient within the 15‐min time frame. Once the patient is evaluated by a provider, the clinical management is at the discretion of the provider in regard to laboratory analysis, medication treatment, frequency of vital sign monitoring and next point of evaluation. There was widespread hospital education around this effort in 2018, and badge cards outlining the process were developed and distributed to all patient care assistants (PCAs), nurses, CNMs and physicians (faculty, residents and fellows) as a visual aid for the process (Figure 1A). A flowsheet in the electronic medical record (EMR) was developed for nursing documentation, and a standardised note template was created for provider documentation. Audits of the EMR were conducted for appropriate documentation and provider responsiveness, beginning after the implementation of MEWS and continuing for 4 years. Random chart audits were also routinely performed to identify any missed MEWS triggers and factors associated with those events. Leaders conducted one‐on‐one coaching and follow‐up for any missed MEWS activations or documentation discrepancies, and patients who triggered MEWS were included in shift reports.

The workgroups that were updating the hospital HTN guideline and developing unit‐based drills/simulations incorporated MEWS throughout the document and training, where applicable, to embed it into the recognition and response for a hypertensive emergency. Activation of MEWS was included in the hypertensive emergency checklist (Figure S1), as were the criteria that defined a hypertensive emergency and treatment recommendations, which formed the basis for the simulations. The updated guideline was disseminated to the department and reviewed at hospital department and committee meetings beginning in February 2022. Following dissemination of the guideline, an order set was developed for the EMR to mirror the hypertensive emergency checklist regarding medication administration and frequency of vital sign monitoring. Additionally, HTN simulation scenarios were scripted with an objective for participants to identify SHTN, active MEWS, recognise a hypertensive emergency and eclamptic seizure, and be able to treat with appropriate medications in a timely manner.

Baseline data was used to inform efforts throughout the development and implementation of the SHTN bundle. MEWS audits were enhanced starting in January 2022 and shifted from auditing all MEWS triggers, as had been done for the preceding 4 years, to auditing only MEWS triggers for SHTN to assess for timely treatment of a hypertensive emergency. To capture automated data on the rate of timely treatment, the hospital's AIM data analyst built a report generated by the EMR to identify all patients who delivered at 20 0/7 weeks or greater that had documentation of a hypertensive emergency. Specifically, the denominator included all birthing patients with acute‐onset SHTN that persisted for 15 min or more, including those with preeclampsia, gestational hypertension, or chronic hypertension. The numerator included all birthing patients in the denominator who had two documented severe BPs within 15–60 min of each other and received treatment within 60 min of the first documented severe BP. Acceptable treatment included any one of the recommended first‐line agents: intravenous labetalol, intravenous hydralazine, or immediate‐release oral nifedipine. These reports became available and were presented in monthly department meetings beginning in June 2022. The hospital quality team then began chart audits of patients who did not receive timely treatment and created a storyboard to illustrate examples of appropriate management and opportunities for improvement (Figure S2). Each storyboard identified a clinical example from three different categories: 1) patient who was treated within 60 min of the first severe BP (displayed in green); 2) patient who did not receive treatment within 60 min of the first severe BP because the BPs became non‐severe (displayed in yellow); and 3) patient who should have received treatment but did not (displayed in red). These storyboards were presented at monthly department and committee meetings attended by nurses and providers. Based on chart audits of patients who did not receive timely treatment, there were several themes identified contributing to the delay. Delayed blood pressure verification was a common theme, particularly in units where BP was automatically cycled every 15–30 min, such as in the obstetric triage unit and labour and delivery. This would subsequently lead to delays in MEWS activation, and hence, delays in provider evaluation and treatment. Another common theme identified was a failure to treat because of intervening non‐severe HTN; however, SHTN would ultimately recur and the treatment was delayed in relation to the first severe BP of that 60 min time frame. These themes allowed the workgroups to target their efforts to mitigate these barriers to timely treatment. In some cases, SHTN would truly resolve without treatment despite the patient meeting criteria for a hypertensive emergency. Patients who had spontaneous resolution of their SHTN were eventually included in the numerator as having received timely treatment based on the definition of a new quality metric for evaluating timely treatment of SHTN from the Society for Maternal‐Fetal Medicine (SMFM) [12]. After this metric was published, our quality team modified our numerator and denominator definitions to be in accordance with the SMFM definition, extended the measure to postpartum patients up to 6 weeks after delivery, and applied the measure to historical data after a rigorous data validation period. Based on our internal data validation process, we made slight adjustments to the SMFM definition (Figure S3), specifically in that we only applied it to pregnancies of at least 20 0/7 weeks gestation, as we found that SHTN episodes prior to 20 0/7 weeks in our hospital were in patients with chronic HTN undergoing inpatient blood pressure medication titration and optimisation with long‐acting agents. We additionally modified our MEWS process to be in accordance with the SMFM definition, specifically that any repeat severe BP obtained within 60 min of the first severe BP was a MEWS activation, even if the severe BPs were not consecutive, rather than a reset of the BP verification process (Figure 1B).

We then conducted a retrospective cohort study around implementation of MEWS and the SHTN bundle to examine whether the interventions influenced an improvement in the rate of timely treatment of a hypertensive emergency in our patient population. We defined phase 1 as our baseline/preintervention (pre‐MEWS) time of January 2017 through May 2018; phase 2 included the period from MEWS implementation (June 2018) up to the start of the TexasAIM SHTN learning collaborative (June 2021); and phase 3 was the period that included implementation and sustainability of the SHTN bundle (June 2021 through December 2024). The primary outcome measure was the rate of timely treatment of a hypertensive emergency. Run and control charts were used to determine if changes in outcomes were true changes and were examined for significance. Due to known disparities in morbidity and mortality associated with hypertensive disorders of pregnancy, our secondary outcomes included racial and ethnic rates of the primary outcome measure as well as rates of spontaneous resolution of SHTN and missed opportunity for treatment (i.e., ‘fallout’). For race and ethnicity classification, our hospital admissions team collects self‐reported race and ethnicity data on all admitted patients and uses the Office of Management and Budget (OMB) Standards [13], with ethnicity being either Hispanic/Latina or NH/Latina and race being categorised as American Indian or Alaska Native, Asian, Black or African American, White, Native Hawaiian or Other Pacific Islander. For purposes of reporting our hospital outcomes, we adopted the following categories: NH‐Black, NH‐White, Hispanic, NH‐Asian and Other. The χ^2^ test was used for categorical outcomes and treatment data were displayed in control and run charts (Minitab V.17.3.1). *p* < 0.05 was considered statistically significant. Since our update to the definition of timely treatment after the SMFM publication resulted in significant changes to our original rates, we report on both sets of values. This study was approved by the Baylor College of Medicine Institutional Review Board for Human Subject Research.

## 3 Results

**FIGURE 2:**
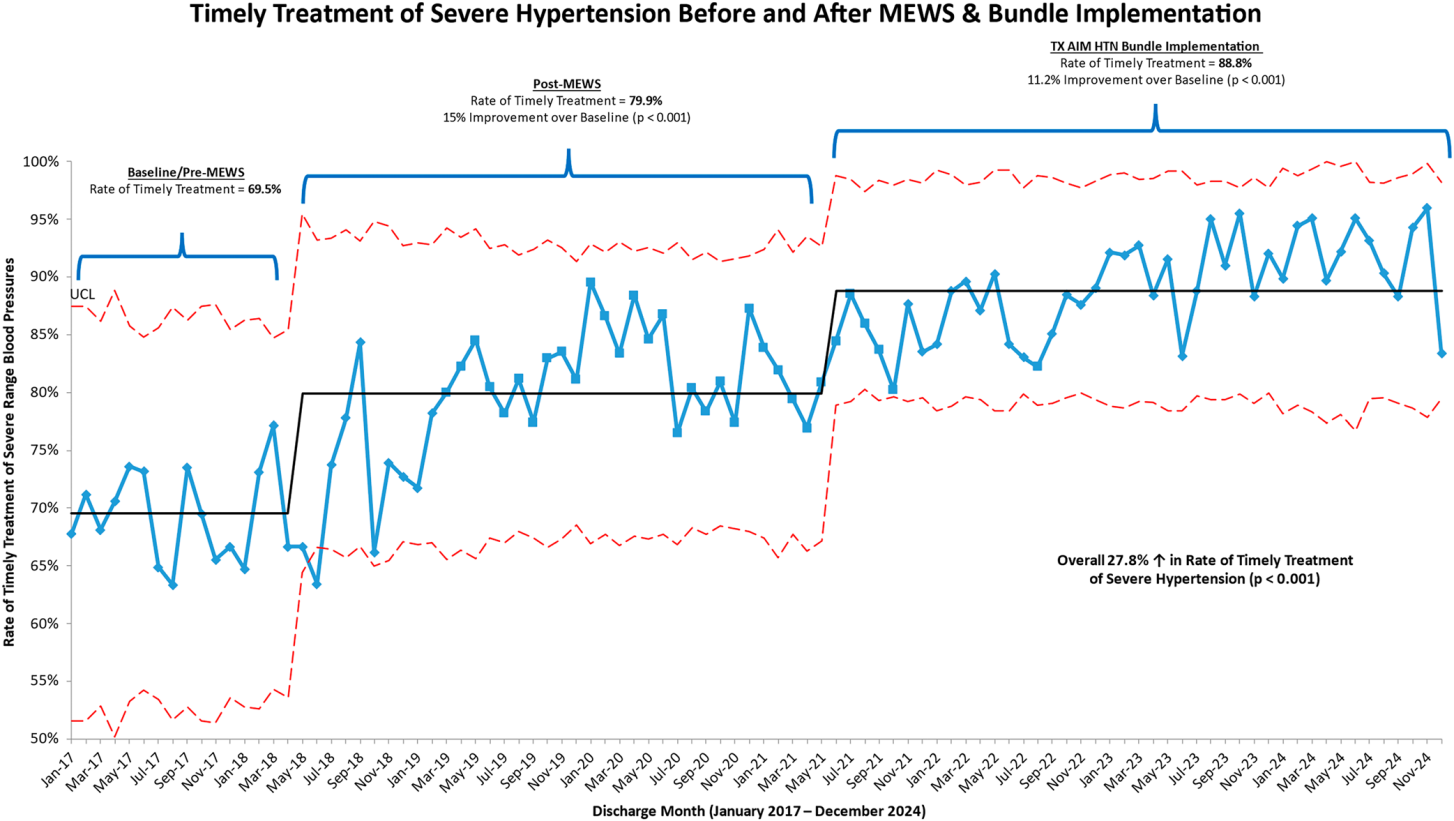
Rate of timely treatment of hypertensive emergency before and after MEWS & bundle implementation. Blue line indicates the rate of timely treatment of hypertensive emergencies from January 2017 through December 2024. Black line represents median (by phase) timely treatment rate. Dotted red line represents the upper control limit and the lower control limit. Phase 1 = Baseline/Pre‐MEWS, Phase 2 = MEWS, Phase 3 = SHTN Bundle.

During the study period, there were 51 585 total deliveries: 8148 deliveries during phase 1 (pre‐MEWS), 20 432 during phase 2 (MEWS) and 23 005 during phase 3 (MEWS plus the SHTN bundle). Patient characteristics over each phase are presented in Table 1. Of the total encounters, 1139 patients met inclusion criteria in phase 1, 3265 in phase 2, and 3952in phase 3. Using the SMFM metric definition, there was a statistically significant improvement in the rate of timely treatment of hypertensive emergencies with implementation of MEWS (69.5% in phase 1 vs. 79.9% in phase 2, *p* < 0.001, 15% improvement) and then again with the bundle (88.8% in phase 3, *p* < 0.001, 11% improvement compared to phase 2 and 27.8% improvement compared to phase 1, *p* < 0.001) (Figure 2). While the original hospital metric also demonstrated a statistically significant improvement in the rate of timely treatment over each time period, the absolute rate (25.9% in phase 1, 44.0% in phase 2, 66.1% in phase 3) was much lower than the rates seen with the SMFM metric (Figure S4).

**TABLE 1:** Maternal characteristics.

Characteristic	Phase 1: Baseline (pre‐MEWS) (*N* = 8148)	Phase 2: MEWS (*N* = 20 432)	Phase 3: MEWS plus HTN bundle (*N* = 23 005)
Age, mean	30.7	30.3	30.6
Delivery gestational age, (median)	39.1	39.0	39.0
BMI at delivery, kg/m^2^ (median)	30.5	31.3	32
Race and ethnicity, *n* (%)			
NH‐White	3244 (39.8%)	6689 (32.7%)	6838 (29.7%)
NH‐Black	1542 (19.0%)	4106 (20.1%)	4801 (21%)
Hispanic	2606 (32.0%)	7947 (38.9%)	9486 (41.2%)
NH‐Asian	668 (8.2%)	1457 (7.1%)	1550 (6.7%)
Other	88 (1%)	233 (1.1%)	330 (1.3%)
Needs Interpreter	456 (5.6%)	1622 (8.1%)	1739 (7.6%)
Nulliparous	1761 (21.6%)	4313 (21.1%)	4563 (19.8%)
Delivery Mode			
Caesarean	2970 (36.5%)	7851 (38.4%)	8996 (39.1%)
Vaginal	5173 (63.5%)	12 573 (61.6%)	14 004 (60.9%)
Insurance			
Commercial	5417 (66.5%)	12 426 (60.8%)	14 330 (62.3%)
Medicaid	2638 (32.4%)	7840 (38.4%)	8491 (36.9%)
Self‐Pay	93 (1.1%)	166 (0.8%)	184 (0.8%)

*Note:* Phase 1: 1/1/2017–5/31/2018. Phase 2: 6/1/2018–6/30/2021. Phase 3: 7/1/2021–12/31/2024.

Abbreviations: BMI, body mass index; NH, non‐Hispanic.

Given the suspected contribution of spontaneous resolution of SHTN into the numerator with the updated SMFM metric, we conducted a review of patient records from April 2022 through February 2023 for those meeting criteria for SHTN during their birth admission with spontaneous resolution within 60 min. Charts were analysed for patient and pregnancy characteristics, hypertension‐related discharge diagnoses, reasons treatment was withheld, rates of SMM, and 30‐day hospital readmission. Our objective was to evaluate the clinical characteristics of those patients that met the timely treatment definition by the SMFM metric and not by our former hospital metric to understand how spontaneous SHTN resolution affected clinical outcomes. Of the 283 patients with a SHTN episode, 46 met inclusion criteria. Of those, 72% had a discharge diagnosis of gestational hypertension or preeclampsia, 2% had chronic HTN and 26% had no HTN‐related diagnosis. Only one patient received a long‐acting anti‐hypertensive agent at any point during their birth admission. The most common documented reason for no treatment was spontaneous resolution of SHTN at the time of physician assessment (41.3%). There were no readmissions or SMM events in the 26% without a hypertensive discharge diagnosis. Of those with a hypertensive discharge diagnosis, three (8.8%) were readmitted for blood pressure complications and none had a non‐transfusion SMM.

Changes over time within each racial and ethnic group indicated that the rate of timely treatment of a hypertensive emergency increased significantly for NH‐Black, NH‐White and Hispanic patients with the implementation of MEWS alone (from phase 1 to phase 2) and for all groups from phase 1 to phase 3 (Table 2). This was observed using both the original hospital metric (data not shown) and the updated SMFM metric definition. Comparing receipt of timely treatment between different racial and ethnic groups at each phase of the study, the original hospital metric demonstrated that NH‐White patients were less likely to receive timely treatment as compared to NH‐Black patients during phases 1 and 2, with elimination of this disparity in phase 3 (*p* < 0.001, 0.04, 0.42, respectively). When the SMFM metric validation process was completed and we applied the updated metric to our historical patient population, however, this finding was no longer observed; by phase 3, NH‐White patients were significantly more likely to receive timely treatment as compared to NH‐Black patients (*p* < 0.001), but similar to everyone else. Additionally, when evaluating the contribution of spontaneous resolution of SHTN for each racial and ethnic group, NH‐Black patients were significantly less likely to have spontaneous resolution (46% vs. 55%) and significantly more likely to experience a treatment fallout (14% vs. 10%, *p* = 0.008) as compared to NH‐White patients, who had the most optimal rates for those measures (Figure S5). After modification of the MEWS process to improve recognition of hypertensive emergencies and facilitate timely treatment (Figure 1B), the fallout rate decreased significantly for both NH‐Black and NH‐White patients (5% for NH‐White, 9% for NH‐Black, *p* = 0.03 for both), the timely treatment rate increased significantly for both groups (from 90.1% to 94.8% for NH‐White patients, from 85.7% to 90.6% for NH‐Black patients, *p* = 0.03 for both groups), and the Black–White disparity for treatment fallouts and timely treatment was eliminated (*p* = 0.06 for both). For calendar years 2023 and 2024, the overall rate of timely treatment ranged from 83% to 96%.

**TABLE 2:** Rate of timely treatment of hypertensive emergency by race and ethnicity.

Race and ethnicity	Phase 1 rate of timely treatment (%)	Phase 2 rate of timely treatment (%)	Phase 3 rate of timely treatment (%)	% improvement from phase 1 to 3
NH‐Black	65.2	79.6*	86.9^#, ^^	33.3
NH‐White	71.1	78.9*	91.3^#, ^^	28.5
Hispanic	69.4	83.0*	89.2^#, ^^	28.5
NH‐Asian	79.4	80.8	88.6^#^	11.6

Abbreviation: NH, non‐Hispanic.

* *p* < 0.05 between phases 1 and 2; ^#^ *p* < 0.05 between phases 2 and 3; ^*p* < 0.05 between phases 1 and 3.

## 4 Discussion

Main Findings: This study demonstrates an association between the implementation of a MEWS and SHTN patient safety bundle and an improved rate of timely treatment of a hypertensive emergency during pregnancy and postpartum. Over the course of implementation, we observed a 15% improvement in the rate of timely treatment from the implementation of MEWS alone and an additional 11.2% improvement with the implementation of a SHTN bundle as compared to MEWS alone, which represented a 27.8% improvement in our rate of timely treatment as compared to our baseline period. Our study also demonstrates an association between a robust MEWS process with the elimination of racial and ethnic disparities in the rate of treatment fallouts for a hypertensive emergency. Additionally, our study illustrates the significant differences in timely treatment rates based on whether or not spontaneous resolution of a SHTN episode is considered timely treatment.

Our findings add to the body of work that highlights the impact of quality and patient safety initiatives aimed at standardising delivery care as well as data disaggregation to allow for hospitals and healthcare systems to become aware of their disparities and work to eliminate them [11, 14, 15]. Our previous work with bundle implementation and data disaggregation also identified racial disparities, with our NH‐Black birthing persons being at highest risk for SMM from obstetric haemorrhage [11]. Our efforts resulted in a reduction in SMM from haemorrhage and elimination of the NH‐Black vs. NH‐White disparity [11]. We attributed our improvements to the implementation of the haemorrhage bundle with a health equity lens and an attempt to mitigate implicit bias in treatment through the standardisation of care and heightened awareness around our disparities. The findings of our current work support that this same approach may improve timely treatment of a hypertensive emergency and eliminate disparities. Based on our original hospital metric definition, our NH‐Black birthing people were more likely to receive timely treatment as compared to our NH‐White birthing people, even before implementation of our quality and patient safety efforts. While this finding was consistent with recent publications demonstrating that White race is associated with a treatment delay for obstetric hypertensive emergencies [16, 17], this was no longer true once spontaneous resolution of SHTN episodes was included in the numerator as the patient having received timely treatment. Investigators have suggested that bias favours expedited treatment in the NH‐Black population given their known increased risks of pregnancy‐related morbidity and mortality from hypertension and preeclampsia, whereas bias may result in delayed treatment of White patients because they do not fit into the mental model of a patient at risk for preeclampsia and its associated complications [16]. Since these studies were published prior to the SMFM quality metric [12], it is possible that spontaneous resolution of SHTN episodes was not considered timely treatment. Our findings suggest that NH‐Black patients are the least likely to have spontaneous resolution of their SHTN episodes and the most likely to have a missed opportunity for timely treatment. Our rigorous data validation and chart audits also provide reassurance that the SMFM quality metric adequately captures patients who may ultimately require treatment for sustained SHTN without missing opportunities to reduce hypertensive morbidity in the group whose SHTN episodes spontaneously resolve.

Interpretation: Our findings also add to the literature on the impact of MEWS. Case reviews of maternal deaths have revealed a pattern of delay in recognition of haemorrhage, hypertensive crisis, sepsis, venous thromboembolism and heart failure, and early‐warning systems have been proposed to facilitate timely recognition, diagnosis and treatment for birthing persons developing critical illness [7]. There are limited data, however, on whether these types of clinical assessment tools can reduce maternal morbidity. Shields and colleagues implemented a maternal early warning tool (MEWT) that was designed to address four of the most common causes of maternal morbidity, as well as provide assessment and management recommendations, and this resulted in significant improvement in maternal morbidity, an outcome measure [18]. Our study reports on improvement of a process measure that is known to be associated with progression to hypertension‐preeclampsia related outcomes. This suggests that monitoring of process measures may be another important factor to consider for reducing preventable pregnancy‐related morbidity and mortality. Our findings also highlight the importance of coupling a MEWS with standardised treatment recommendations for MEWS triggers, as done in the MEWT study, as our rate of timely treatment peaked once the goals of timely treatment were incorporated into our MEWS response. Prior to implementation of the SHTN bundle, clinical care following a MEWS trigger was at the discretion of the individual provider.

The objectives of treating a hypertensive emergency are to prevent congestive heart failure, myocardial ischemia, renal injury or failure, and ischemic or haemorrhagic stroke. The available literature suggests that antihypertensive agents should be administered within 30–60 min of a hypertensive emergency; however, it is recommended to administer antihypertensive therapy as soon as reasonably possible after the criteria for acute‐onset SHTN are met [5]. Given the finding from our internal audits that some of our delays were occurring in patients with non‐severe HTN interspersed between SHTN values, we recognised an area of our MEWS process that required optimisation. Through interviews with clinical nurses, we learned that MEWS was not activated unless the severe‐range BPs were consecutive; if another severe BP was recorded after a non‐severe BP, the process of BP verification would restart. This led to the update in our MEWS process as illustrated in Figure 1B, which proved to have a significant impact on improving our rate of timely treatment and decreasing our fallout rate. Our study illustrates some approaches to improving the rate of timely treatment of a hypertensive emergency of pregnancy; however, additional measures may be necessary to further improve our rate. Implementation of a semiautonomous treatment algorithm for SHTN has been shown to be associated with a higher percentage of pregnant and postpartum patients receiving the first dose of antihypertensive therapy within 15 and 30 min, and no hypotensive episodes were observed during the study period. The investigators suggest that implementation of similar algorithms for this and other obstetric indications may decrease time to appropriate therapy and help improve care equity [19].

Strengths and Limitations: Our study is limited in that our process measure only captures the first severe BP and does not account for recurrent hypertensive emergencies in the same patient. Our process measure, however, is endorsed by SMFM and AIM [6, 8]. Our study has several notable strengths. We describe a series of quality improvement processes that can be replicated at any hospital to improve the rate of timely treatment of a hypertensive emergency and reduce racial and ethnic disparities. While the National Institute for Health and Care Excellence (NICE) guidelines on hypertension in pregnancy do not specify a time limit by which to treat SHTN, they align with SMFM and ACOG recommendations in that women with SHTN who are in critical care during pregnancy or after birth should be treated immediately with labetalol (oral or intravenous), oral nifedipine, or intravenous hydralazine [20], thus making our interventions broadly applicable to any guideline recommendation on the treatment of SHTN. We also demonstrate the importance of stratifying process measures, and not just outcome measures, by race and ethnicity to identify disparities that could be contributing to the disparities in outcomes. This allows for situational awareness of the impact that implicit bias may have on patient care and allows teams to target improvement in process measures as a means to improving overall outcomes.

## 5 Conclusion

Our study suggests that the use of a MEWS combined with implementation of the AIM SHTN patient safety bundle improves early recognition and response to an obstetric hypertensive emergency and reduces racial and ethnic disparities. This may lead to a reduction in preventable morbidity and mortality from hypertensive disorders of pregnancy. Our findings also suggest that the adoption of the SMFM quality metric definition for timely treatment of SHTN, which considers spontaneous resolution of SHTN as timely treatment and captures patients with hypertensive emergencies who have intervening non‐SHTN, will more appropriately identify patients who would benefit from acute‐acting anti‐hypertensive medication.

## Author Contributions

Towana D. Sims and Christina Davidson are primary writers and implementers of the quality improvement initiative. Lauren Shubert, Stacie Denning, Celeste Green and Lena Shay are the primary chart abstractors and data analyzers, who contributed to the results.

## Conflicts of Interest

The authors declare no conflicts of interest.

## Supporting information


Data S1.

## Data Availability

The data that support the findings of this study are available from the corresponding author upon reasonable request.
