# Supplementary material for: Impact of a Maternal Early Warning System and Severe Hypertension Safety Bundle on Timely Treatment of Hypertensive Emergencies: A Quality Improvement and Health Equity Initiative
Source: BJOG. 2025 Jul 17;132(13):2160–7. doi: 10.1111/1471-0528.18273 (PMC12592757; doi:10.1111/1471-0528.18273)

**Figure S1.** Hypertensive Emergency Checklist

Figure S1 Checklist includes recognition signs, medication/treatment protocols and escalation guidance.


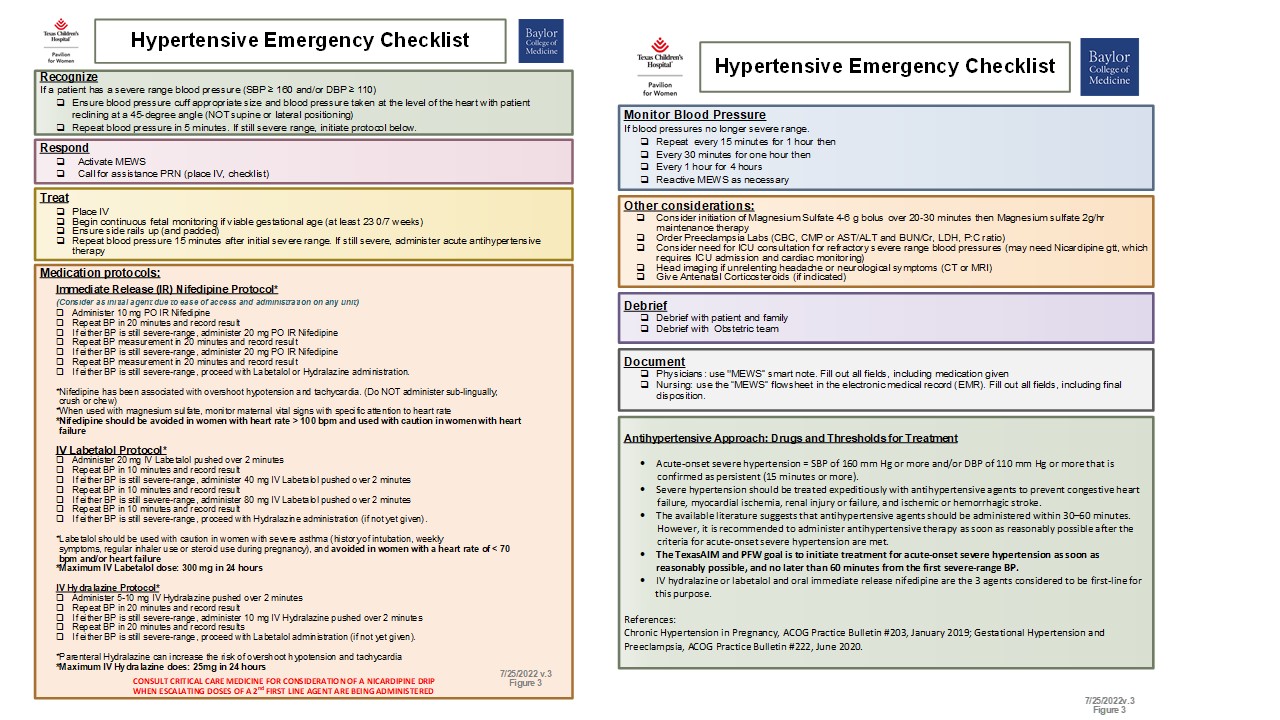


**Figure S2**. Timeliness of Treatment Audit

Figure S2 is an example of the storyboard that was shared with the women’s services department to illustrate clinical overview surrounding the hypertensive emergency episode and opportunities for improvement. Patient treated within 60 minutes of the first severe BP (displayed in green). Patient who did not receive treatment within 60 minutes of the first severe BP because the BPs became non-severe spontaneously (displayed in yellow). Patient that should have received treatment but did not (displayed in red).


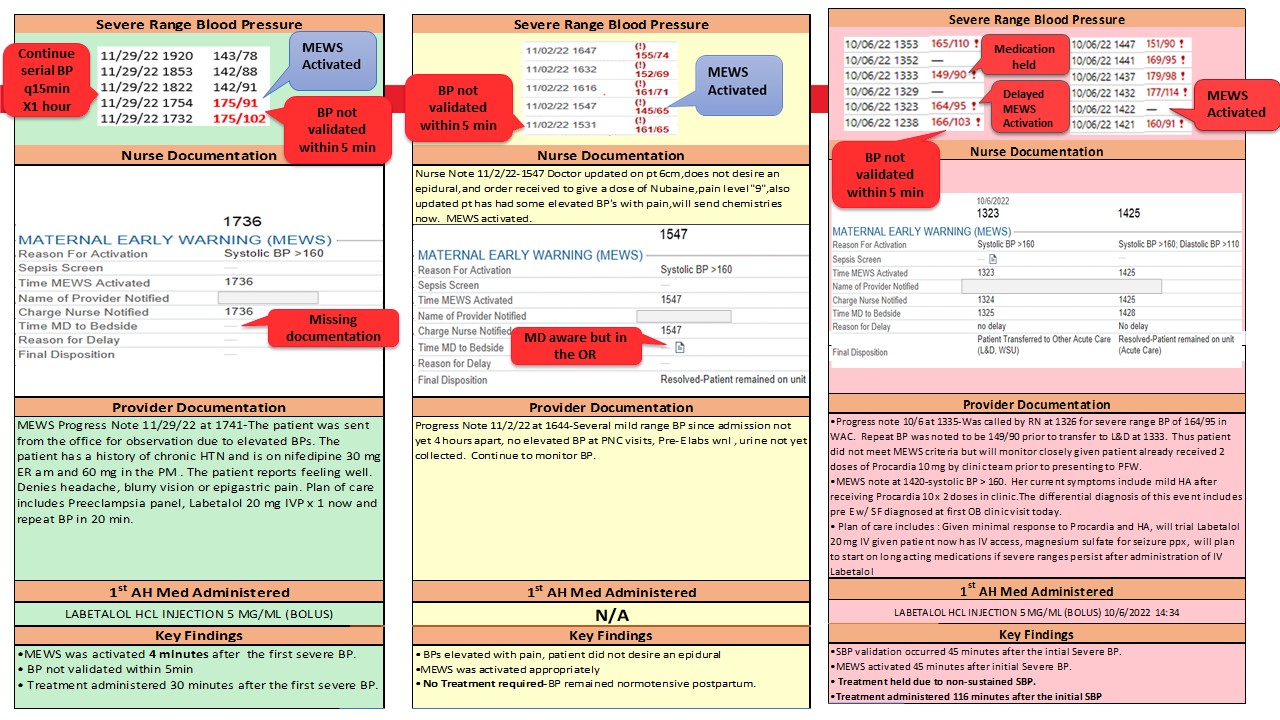


**Figure S3:** Quality Metric for Timely Treatment of Severe Hypertension – based on SMFM metric

Figure S3 is the logic algorithm that was used to abstract data reports from the electronic medical record for the updated Society for Maternal-Fetal Medicine metric on timely treatment of severe hypertension


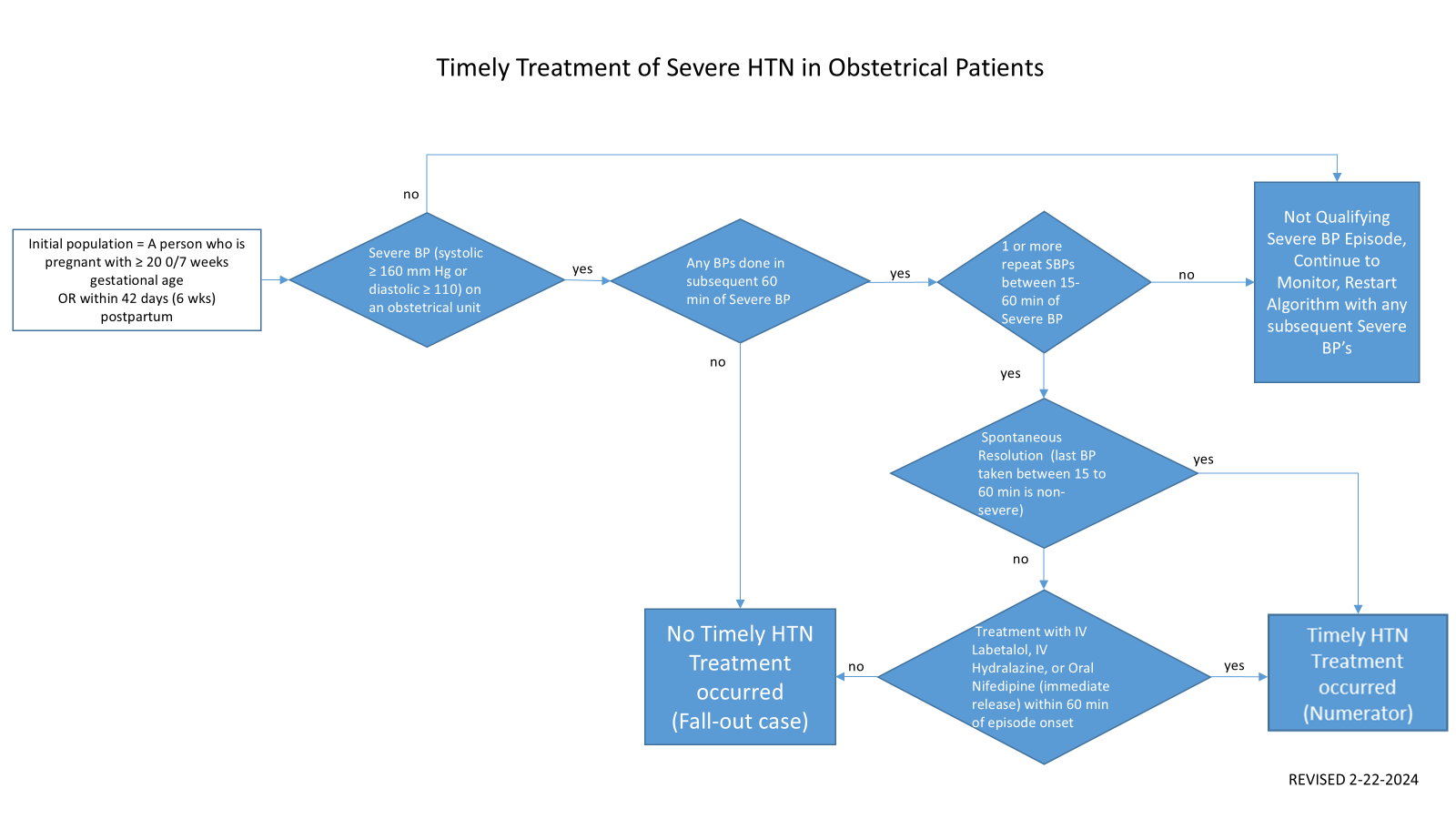


**Figure S4.** Rate of Timely Treatment of Hypertensive Emergency

**
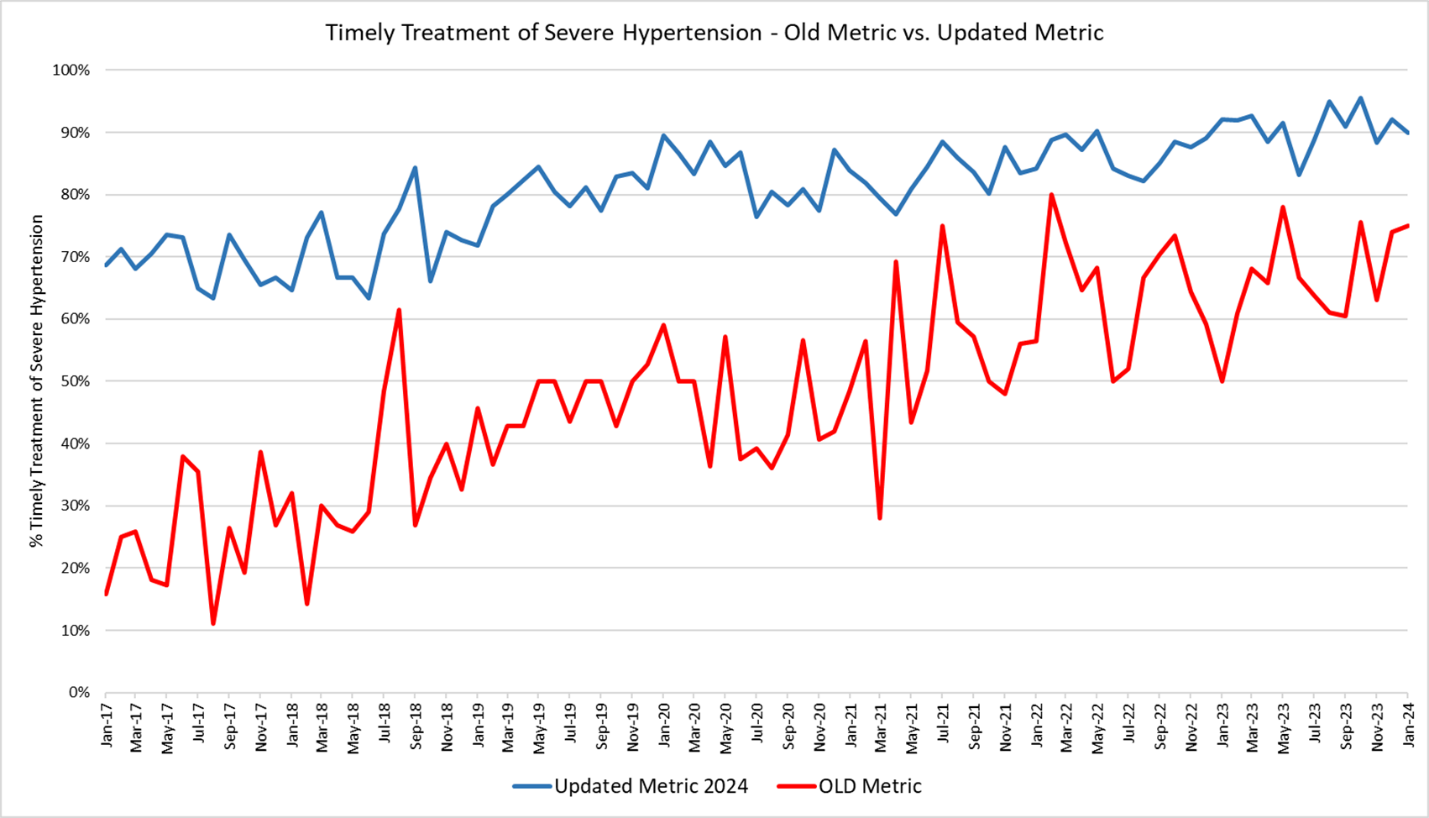
**

Red line indicates the rate of timely treatment of severe range blood pressures from January 2017 through January 2024 based on the original hospital metric, which only included the delivery admission in the denominator and did not include spontaneous resolution in the numerator

Blue line indicates the rate of timely treatment of severe range blood pressures from January 2017 through January 2024 based on the updated hospital metric in accordance with the SMFM definition

**Figure S5.** Rate of Timely Treatment vs. Fallouts vs. Spontaneous Resolution of Hypertensive Emergency by Race and Ethnicity Group, Comparison of 2 different MEWS policies

Figure S5 illustrates the rate of timely treatment of a hypertensive emergency, rate of spontaneous resolution of a hypertensive emergency, and rate of missed opportunity for treatment (fallout) for a hypertensive emergency by race and ethnicity. The “old MEWS” graph represents these rates prior to updates to the MEWS process. The “new MEWS” process reflects the rates after the policy was updated to activate a MEWS response for any severe blood pressure obtained within 60 minutes of the first severe blood pressure, even if interspersed with non-severe blood pressures.


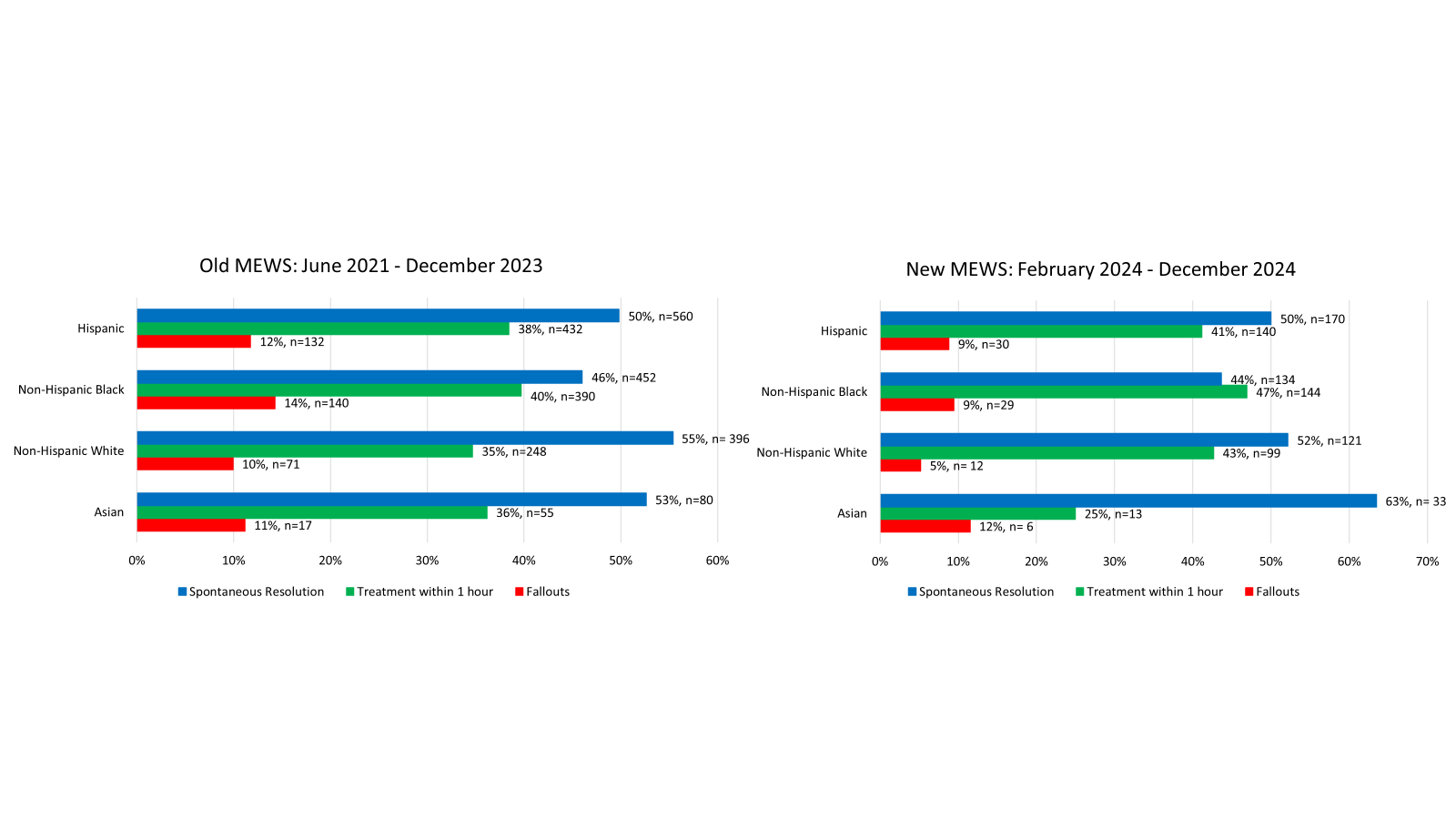

Supplement: Supplementary file 1 — Data S1. [file BJO-132-2160-s001.docx]
